# Longitudinal Changes of One-Carbon Metabolites and Amino Acid Concentrations during Pregnancy in the Women First Maternal Nutrition Trial

**DOI:** 10.1093/cdn/nzz132

**Published:** 2019-11-18

**Authors:** Stephanie P Gilley, Nicholas E Weaver, Evan L Sticca, Purevsuren Jambal, Alexandra Palacios, Mattie E Kerns, Pratibha Anand, Jennifer F Kemp, Jamie E Westcott, Lester Figueroa, Ana Lucía Garcés, Sumera A Ali, Omrana Pasha, Sarah Saleem, K Michael Hambidge, Audrey E Hendricks, Nancy F Krebs, Sarah J Borengasser

**Affiliations:** 1 Department of Pediatrics, Section of Nutrition, University of Colorado Anschutz Medical Campus, Aurora, CO, USA; 2 Mathematical and Statistical Sciences, University of Colorado Denver, Denver, CO, USA; 3 Human Medical Genetics and Genomics Program, University of Colorado Anschutz Medical Campus, Aurora, CO, USA; 4 Institute of Nutrition in Central America and Panama, Guatemala City, Guatemala; 5 Aga Khan University, Department of Community Health Sciences, Karachi, Pakistan; 6 Department of Population, Family and Reproductive Health, Johns Hopkins University Bloomberg School of Public Health, Baltimore, Maryland, USA

**Keywords:** one-carbon metabolism, amino acids, preconception, pregnancy, BMI, obesity, malnutrition, supplementation, triple nutrition burden

## Abstract

**Background:**

Maternal dietary restriction and supplementation of one-carbon (1C) metabolites can impact offspring growth and DNA methylation. However, longitudinal research of 1C metabolite and amino acid (AA) concentrations over the reproductive cycle of human pregnancy is limited.

**Objective:**

To investigate longitudinal 1C metabolite and AA concentrations prior to and during pregnancy and the effects of a small-quantity lipid-based nutrition supplement (LNS) containing >20 micronutrients and prepregnancy BMI (ppBMI).

**Methods:**

This study was an ancillary study of the Women First Trial (NCT01883193, clinicaltrials.gov) focused on a subset of Guatemalan women (*n* = 134), 49% of whom entered pregnancy with a BMI ≥25 kg/m^2^. Ninety-five women received LNS during pregnancy (+LNS group), while the remainder did not (−LNS group). A subset of women from the Pakistan study site (*n* = 179) were used as a replication cohort, 124 of whom received LNS. Maternal blood was longitudinally collected on dried blood spot (DBS) cards at preconception, and at 12 and 34 wk gestation. A targeted metabolomics assay was performed on DBS samples at each time point using LC-MS/MS. Longitudinal analyses were performed using linear mixed modeling to investigate the influence of time, LNS, and ppBMI.

**Results:**

Concentrations of 23 of 27 metabolites, including betaine, choline, and serine, changed from preconception across gestation after application of a Bonferroni multiple testing correction (*P* < 0.00185). Sixteen of those metabolites showed similar changes in the replication cohort. Asymmetric and symmetric dimethylarginine were decreased by LNS in the participants from Guatemala. Only tyrosine was statistically associated with ppBMI at both study sites.

**Conclusions:**

Time influenced most 1C metabolite and AA concentrations with a high degree of similarity between the 2 diverse study populations. These patterns were not significantly altered by LNS consumption or ppBMI. Future investigations will focus on 1C metabolite changes associated with infant outcomes, including DNA methylation. This trial was registered at clinicaltrials.gov as NCT01883193.

## Introduction

A growing number of low- and middle-income countries suffer from the triple malnutrition burden of undernutrition (including stunting), obesity, and micronutrient deficiencies (1, 2). Poor nutrition is a major contributing factor as low- and middle-income countries undergo a “nutrition transition” toward a more Westernized diet, with resultant increases in obesity rates (3, 4). In particular, Guatemalan households have been reported to have the highest worldwide prevalence of concurrent maternal obesity with offspring stunting and only small population improvements over time (4, 5).

Pregnant women with obesity, undernutrition, and/or micronutrient deficiencies expose their embryos and fetuses to these conditions, which are known to impart increased risk to the offspring for obesity, cardiovascular disease, and diabetes in later life (6–10), a process commonly referred to as the developmental origins of health and disease hypothesis (8, 11). Several epidemiological studies have shown that maternal nutritional status plays a particularly important role at the time of conception and during pregnancy, including maternal exposures during the Dutch famine, seasonal availability of food in The Gambia, obesity, and other forms of malnutrition (7, 8, 11, 12). Interventions targeting maternal nutrition have the potential to improve pregnancy and offspring outcomes (13).

Epigenetic changes are a leading candidate for understanding the link between in utero nutrition exposures and long-term infant health (11, 12, 14, 15). More specifically, maternal nutrition is known to influence the offspring epigenome through the one-carbon (1C) metabolism pathway, as intake of methyl donors and cofactors is required for DNA methylation (12, 15–18). Increased demand for nutrients that serve as methyl donors and cofactors, such as folate, choline, and vitamins B6 and B12, occurs during pregnancy (12, 19, 20). In addition, the essential amino acid (AA) methionine, derived from both dietary and cellular sources, serves as the primary methyl donor for DNA methylation (6, 11, 19, 20), and there is emerging evidence that lipids can influence expression of genes involved in 1C metabolism (21, 22). Further, maternal 1C pathway metabolites and AA concentrations have been associated with fetal growth, making their study important for understanding and preventing stunting (20, 23–25).

Dietary intake and supplementation can influence concentrations of several 1C metabolites and AA during pregnancy (26–28). In addition, some 1C metabolites can serve as biomarkers prior to the presentation of clinical manifestations. For example, betaine concentrations are lower in the second trimester in women who later develop gestational diabetes (29), and homocysteine and asymmetric dimethylarginine (ADMA) are elevated prior to diagnosis of pregnancy hypertensive disorders (30).

Only a few studies have assessed temporal changes in 1C metabolite and AA concentrations during pregnancy (31–34). Of these, most did not longitudinally assess women from prior to conception through pregnancy, nor were possible effects of additional nutrient supplementation or prepregnancy BMI (ppBMI) considered. The present study prospectively measured 1C metabolite and AA concentrations in Guatemalan women prior to conception through late pregnancy as part of the Women First Preconception Maternal Nutrition Trial (Women First Trial) (35, 36). We further compared women receiving a small-quantity lipid-based nutrition supplement (LNS) containing >20 micronutrients, including folate and several B vitamins involved in 1C metabolism, to those following local standard-of-care medical guidelines. We hypothesized that 1C metabolite and AA concentrations in maternal blood would vary by time, and that these trajectories would be further influenced by LNS and ppBMI. Understanding the natural course of these metabolites in the same women over the course of pregnancy and how they are influenced by nutritional supplements and ppBMI may help inform future studies involving supplementation with methyl donors during pregnancy.

## Methods

### Study design and intervention

This study was an ancillary study of a large, randomized controlled trial called the Women First Trial (clinicaltrials.gov NCT01883193), which hypothesized that the timing of maternal LNS consumption (Nutriset) would influence birth length in low-resource communities (35, 36). LNS composition (**Supplemental Table 1**) has been previously described and includes protein, polyunsaturated fatty acids, a favorable ω-3 to ω-6 fatty acid ratio, and >20 micronutrients, including 400-μg folate, 2.8-mg riboflavin, 5.2-μg vitamin B12, and 3.8-mg vitamin B6 (35, 36). After enrollment, subjects (*n* = 7387) were randomly assigned to 1 of 3 study arms: *1*) consumed LNS starting at least 3 mo prior to conception until delivery; *2*) consumed LNS starting at 12 wk of gestation until delivery; or *3*) followed the local standard of care without LNS consumption (35). Women in the intervention arms were additionally given a lipid-based protein energy supplement if they had a BMI <20 kg/m^2^ at any time while receiving LNS, or if their gestational weight gain was below the Institute of Medicine's recommendations (35, 36). The study sites for the trial were Guatemala, Pakistan, Democratic Republic of the Congo, and India. The Women First Trial study protocol was approved by the University of Colorado Institutional Review Board. In addition, ethics committees and local Ministries of Health granted approval at each research site. Women were enrolled in the trial and provided informed consent in accordance with the principles described in the Declaration of Helsinki of 1975 as revised in 1983.

**FIGURE 1:**
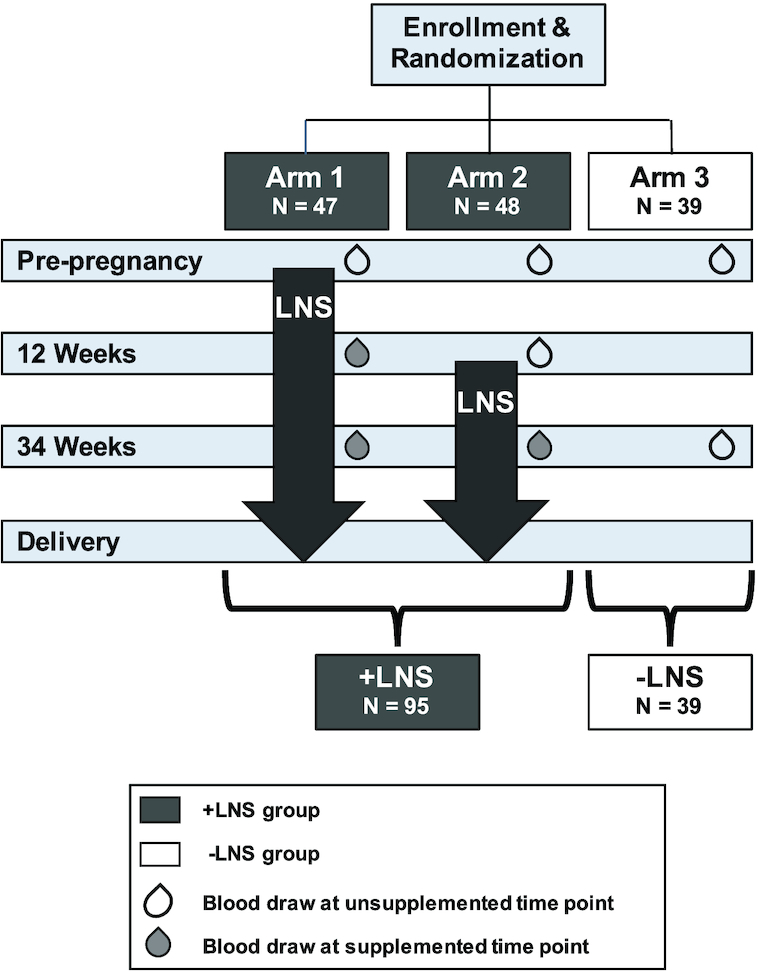
Study and analysis design schematic. At study enrollment women were randomized into 1 of 3 study arms. For the present analysis, blood draws that occurred when the subjects were not consuming LNS (represented by unfilled blood drops) were considered unsupplemented time points; blood draws that occurred when the subjects had taken LNS for at least 12 wk (represented by solid blood drops) were considered supplemented time points. Arms 1 and 2 were combined and represent the +LNS group, while Arm 3 comprised the −LNS group. LNS, small-quantity lipid-based nutrition supplement.

The present study focused on a subcohort of rural Guatemalan mothers (*n* = 134). Subjects were assigned to 2 groups, those who consumed LNS at any time during pregnancy, whether initiated prior to conception or at 12 wk of gestation (+LNS; *n* = 95), compared to those who did not (−LNS; *n* = 39). The study design is shown in **Figure 1**. A replication cohort was selected from the Pakistan study site (+LNS: 124 women; −LNS: 55 women). Subjects were excluded from this ancillary study if they did not conceive and deliver an infant during the course of the larger trial. Subjects were chosen to ensure that LNS groups within each study site were well matched for age, parity, ppBMI, height, and socioeconomic status (SES) (**Table 1**). Maternal height and weight were obtained during an in-person study visit by on-site study personnel who were trained to measure subject anthropometrics using an electronic scale and adult stadiometer. Preconception weight status was defined as underweight (BMI: <18.5 kg/m^2^), normal weight (BMI: 18.5–24.9 kg/m^2^), overweight (BMI: 25–29.9 kg/m^2^), or obese (BMI: >30 kg/m^2^). The SES indicator score was calculated as a score from 0 to 6, with 6 indicating the highest SES status (36). Each SES indicator equaled 1 and all were tallied together to generate the SES indicator score. SES indicators included: (1) electricity, (2) improved water source, (3) sanitation, (4) man-made flooring, (5) improved cooking fuels, and (6) household assets (possessing >1 television, telephone, bike, motorized bike, or scooter, or owns a car or truck).

**TABLE 1:** Subject characteristics for the primary Guatemalan cohort and for the Pakistani replication cohort^1^

	Guatemala (primary cohort)				Pakistan (validation cohort)			
Characteristic	Overall	−LNS	+LNS	*P* value	Overall	−LNS	+LNS	*P* value
*n* (%)	134	39 (29.1)	95 (70.9)		179	55 (30.7)	124 (69.3)	
Maternal age at enrollment, yr	23.4 (16–35)	24.1 ± 0.8	23.4 ± 0.5	0.400	23.2 (16–32)	23.2 ± 0.5	23.2 ± 0.4	0.976
Maternal age at delivery, yr	24.5 (17–36)	25.6 ± 0.8	24.8 ± 0.5	0.384	24.1 (17–33)	23.9 ± 0.5	24.2 ± 0.4	0.676
BMI at enrollment, kg/m^2^	25.2 (18.8–38.4)	26.1 ± 0.7	25.7 ± 0.4	0.624	19.1 (13.8–32.1)**	19.1 ± 0.3^^^^	19.1 ± 0.3^‡^	0.946
Underweight	0	0	0	—	92 (51.4)**	27 (49.1)^^^^	65 (52.4)^‡^	0.681
Normal weight	68 (50.7)	21 (53.8)	47 (49.5%)	0.646	82 (45.8)	27 (49.1)	55 (44.4)	0.557
Overweight/obese	66 (49.3)	18 (46.2)	48 (50.5)	0.646	5 (2.8)**	1 (1.8)^^^^	4 (3.2)^‡^	0.578
Maternal height, cm	149.9 (132.2–158.6)	145.6 ± 0.7	146.1 ± 0.6	0.602	152.8 (134–178)**	152.9 ± 0.8^^^^	152.8 ± 0.6^‡^	0.894
Improved SES score	3.3 (1–6)	3.9 ± 0.1	3.8 ± 0.1	0.605	3.0 (0–6)**	2.9 ± 0.2^	3.0 ± 0.1^‡^	0.654
Parity	1.6 (0–5)	1.8 ± 0.2	1.6 ± 0.1	0.202	1.6 (0–5)	1.6 ± 0.2	1.5 ± 0.1	0.697
Vaginal delivery	74 (55.2)	22 (56.4)	52 (54.7)	0.860	162 (90.5)**	50 (90.9)^^^^	112 (90.3)^‡^	0.626
Female infant	457 (42.5)	16 (41.0)	41 (43.2)	0.821	96 (53.4)*	25 (45.5)	71 (57.3)^†^	0.177
Compliance with LNS	—	—	73.4 (30.4–99.1)	—	—	—	87.0 (43-100)^‡^	—
Total days on LNS	—	—	346 (139–747)	—	—	—	356.9 (139–786)	—
Receiving calorie supplement	—	—	6 (6.3)	—	—	—	118 (95.2)^‡^	—
Taking folate supplement at baseline	28 (20.9)	20.5 (8)	21.1 (20)	0.944	0**	0^^^	0^‡^	—
Taking folate supplement at 12 wk	41.0 (55)	69.2 (27)	29.5 (28)	*<0.0001*	2.8 (5)**	5.5 (3)^^^^	1.6 (2)^‡^	0.150
Taking folate supplement at 34 wk	30.6 (41)	84.6 (33)	8.4 (8)	*<0.0001*	8.9 (16)**	23.6 (13)^^^^	2.4 (3)^†^	*<0.0001*

1 Values are means (ranges), percentages (*n*), or means ± SEMs unless otherwise indicated. Significant differences (*P* < 0.05, indicated in *italics*) , between −LNS and +LNS groups within each study site and between study sites were determined by unpaired *t* test for continuous variables and chi-square test for categorical variables. Symbols denote significant differences between the Guatemalan and Pakistani cohorts overall, *P < 0.05; **P < 0.0001; between −LNS groups, ^^^*P* < 0.05; ^^^^*P* < 0.0001; and between +LNS groups, ^†^*P* < 0.05; ^‡^*P* < 0.0001. LNS, small-quantity lipid-based nutrition supplement; SES, socioeconomic status.

LNS compliance was assessed by subject self-report through daily calendars as well as by collection of empty and unused intervention sachets by on-site study personnel (35, 36). Any additional folate supplementation was self-reported by subjects at study enrollment, 12 wk, and 34 wk. Frequency was reported as daily, weekly, or monthly. After delivery, study personnel recorded whether the woman had any hypertension during pregnancy (including gestational hypertension, preeclampsia, and eclampsia). Specific vital sign values and the results of any clinical tests such as urine protein or platelet count were not recorded.

### Blood collection and 1C metabolite/AA profiling using dried blood spot cards

Blood samples were collected at study enrollment, 12 wk gestation, and 34 wk gestation by trained study personnel (**Figure 1**). Women were not fasting at the time of blood draw. Approximately 0.5 mL of whole blood was applied to a Whatman 903 protein saver dried blood spot (DBS) card (GE Healthcare Life Sciences) and dried for at least 4 hours. DBS cards were then stored at − 20°C with desiccant packs and humidity indicator cards.

A targeted quantitative 1C and AA analysis panel was performed using LC-MS/MS at the Southeast Center for Integrated Metabolomics at the University of Florida. The standard panel consisted of 36 amino acids, methylated amino acids and 1C metabolites. From this panel, the light-sensitive metabolites S-adenosylmethionine and S-adenosylhomocysteine were excluded as the DBS cards were not specifically protected from light. Briefly, two 3-mm punches from DBS samples collected at all 3 study time points were used for 2 assays to determine the 1C metabolite and AA concentrations. For the 1C–AA assay, blood spots were rehydrated with water and internal standard mix. After sonication, acetonitrile was added to precipitate protein, the sample was vortexed, and sonicated again (37). For the thiol assay, DBSs were rehydrated with water, internal standard mix, and BondBreaker (ThermoFisher Scientific). After sonication, a fresh preparation of 100 mM iodoacetic acid in 200 mM ammonium bicarbonate/200 mM ammonium hydroxide was added. After this mixture was incubated in the dark, acetonitrile was added, and then the sample was vortexed and sonicated again (38). All samples from both assays were then centrifuged (2500 × *g* for 10 min at 5°C) , and the supernatant was transferred to a 96-well plate for analysis by LC-MS/MS. Samples were chromatographed on a Waters Cortecs HILIC column and eluted with an acetonitrile–water gradient containing ammonium formate and formic acid. Mass spectrometric detection was on a Bruker EvoQ Elite MS/MS (Billerica) in positive ion mode, using a heated electrospray ionization source. For metabolite quantitation, authentic isotopically labeled forms of each metabolite were used as internal standards. Peak area ratios were calculated by dividing the metabolite peak area by the peak area of its internal standard. Metabolite concentrations were calculated by comparing these peak area ratios to standard curves prepared using authentic standards.

### Quality control and statistical analysis

Subjects were grouped by LNS status instead of according to their original Women First Trial arm because *1*) biological samples were not collected from Arm 3 subjects at 12 wk of gestation, due to trial study design (Figure 1) (35); and *2*) because change over pregnancy was of primary interest. Subjects in Arm 2 had blood drawn at 12 wk gestation, which was prior to supplementation; thus, the study groupings allowed for Arm 2 subjects to fill in the sample size at the 12-wk time point to allow LNS status differences to be tested. Metabolites with missing concentration measurements for >10% of samples were removed from further analysis. Following natural log transformation, metabolite concentrations were normalized within each experimental batch by subtracting off the mean log(metabolite concentration) for prepregnancy values within each batch, e.g., log(12 wk) – [mean log(preconception)]. This resulted in a within-batch mean of zero at preconception and change from prepregnancy for other time points. Statistical outliers were defined as 1.5 times the IQR below or above the first or third quartiles, respectively. All analyses were run with and without outliers to assess robustness of the results. Individuals with missing metabolite values were excluded only from the analysis for which they were missing data.

Significant differences in subject characteristic covariates between −LNS and +LNS groups within and between each study site were determined by unpaired *t* test for continuous variables and by chi-square test for categorical variables. Linear mixed effect (LME) models with a random intercept for the individual subject were used to assess the relation between each metabolite and time, controlling for geographic cluster (i.e., region in Guatemala or Pakistan where women were recruited to the study), LNS status at the time of blood sampling, and ppBMI. Time (i.e., preconception, 12 wk, or 34 wk) was assessed as a categorical variable, and ppBMI was assessed as a continuous variable. Potential interactions between LNS status and time as well as ppBMI and time were assessed. Interactions were removed from the final LME models as they were not significant (i.e., *P* > 0.05). Results for the relations of metabolite concentrations with ppBMI, LNS status, and time are reported from the model without interaction terms. Significance was assessed using a conservative Bonferroni correction for 27 metabolites (*P* < 0.05/27 = 0.00185) in the Guatemalan cohort. Replication in the Pakistani cohort was assessed as having effects in the same direction as the Guatemalan cohort at a nominal significance threshold of *P* < 0.05 as well as the more conservative Bonferroni threshold of *P* < 0.00185. Adjusted *P* values and 95% CIs for ad hoc pairwise comparisons of time of blood sampling were calculated using Tukey's adjustment. Compliance was not considered part of the statistical model, consistent with use of a modified intention-to-treat analysis for other study outcomes (36).

## Results

### Subject characteristics

A total of 134 Guatemalan women were included in this ancillary study (Table 1), in which 29% of women did not consume LNS and 71% of women did. Of the +LNS group, 49.5% (*n* = 47) started LNS at least 3 mo prior to conception and the remainder (*n* = 48) initiated LNS at 12 wk of gestation. Six women in the +LNS group received an additional calorie supplement due to inadequate gestational weight gain. Subjects were well matched for several maternal characteristics that included age, parity, height, ppBMI, SES, mode of delivery, and infant sex, as shown in Table 1. Women assigned to the −LNS group were advised to follow local medical care guidelines regarding supplementation with nutrients, including folate. At 34 wk, 84.9% of the women in the −LNS group reported taking folate at least weekly. Women assigned to the intervention were asked to abstain from taking any nonstudy supplements; however, some women did report taking additional folate (Table 1). At study enrollment, 49% of the 134 women were overweight (*n* = 44) or obese (*n* = 22). The remaining 68 women had a normal ppBMI.

### 1C metabolite levels

Seven metabolites (carnitine, deoxycarnitine, glutamate, glycine, homocysteine, leucine, and methionine sulfoxide) had missingness >10% and were removed from further analysis. Twenty-three of the 27 remaining metabolites significantly changed over the course of pregnancy (**Table 2**). Only ADMA, arginine, glutathione, and methionine did not statistically change from preconception through 34 wk of pregnancy. The concentrations of 2 metabolites, ADMA and symmetric dimethylarginine (SDMA), were decreased at 34 wk in the +LNS group (**Supplemental Figure 1**). Temporal patterns were not altered by ppBMI, as the removal of ppBMI from the statistical model did not result in considerable change in the relations of time and concentration for any metabolite. The concentrations of 3 metabolites—homoarginine, tyrosine, and valine—were positively associated with ppBMI (Table 2). All results were consistent when outliers were included compared with when they were excluded.

**TABLE 2:** Fold change of log-transformed metabolite concentrations over time with *P* values in Guatemalan primary cohort^1^

				Baseline to 12 wk		Baseline to 34 wk		12 to 34 wk	
Metabolite	Overall supplement *P* value	Overall time *P* value	Overall ppBMI *P* value	Fold change (95% CI)	*P* value	Fold change (95% CI)	*P* value	Fold change (95% CI)	*P* value
ADMA	*6.54E-04*	0.395	0.161	0.95 (0.86, 1.04)	0.382	0.98 (0.89, 1.07)	0.807	1.03 (0.95, 1.11)	0.722
Alanine	0.155	*2.68E-12*	0.066	0.85 (0.79, 0.91)	*8.11E-08*	0.80 (0.74, 0.86)	*2.94E-12*	0.94 (0.89, 1.00)	0.048
Arginine	0.418	0.173	0.886	0.97 (0.85, 1.11)	0.890	1.07 (0.93, 1.22)	0.512	1.09 (0.98, 1.23)	0.158
Betaine	0.870	*<1.00E-16*	0.694	0.46 (0.43, 0.50)	*3.46E-14*	0.36 (0.33, 0.39)	*<1.00E-16*	0.78 (0.73, 0.84)	*1.34E-13*
Choline	0.614	*5.39E-04*	0.690	1.15 (0.94, 1.41)	0.236	0.86 (0.69, 1.06)	0.200	0.74 (0.62, 0.89)	*3.65E-04*
Citrulline	0.059	*<1.00E-16*	0.399	0.67 (0.62, 0.73)	*1.72E-14*	0.60 (0.56, 0.65)	*1.78E-13*	0.90 (0.84, 0.96)	*7.61E-04*
Creatine	0.732	*2.22E-16*	0.003	1.05 (0.99, 1.12)	0.151	1.43 (1.33, 1.54)*	*3.14E-14*	1.36 (1.28, 1.44)*	*1.96E-13*
Creatinine	0.029	*<1.00E-16*	0.635	0.77 (0.73, 0.81)	*1.71E-14*	0.78 (0.74, 0.83)	*1.95E-13*	1.01 (0.97, 1.06)	0.800
Cysteine (total)	0.066	*2.26E-06*	0.351	1.00 (0.90, 1.12)	0.998	0.82 (0.73, 0.92)	*2.53E-04*	0.82 (0.75, 0.90)	*6.22E-06*
Glutamine	0.735	*1.49E-04*	0.323	0.88 (0.82, 0.95)	*4.76E-04*	0.88 (0.81, 0.95)	*5.06E-04*	0.99 (0.93, 1.06)	0.976
Glutathione (total)	0.869	0.072	0.370	1.06 (0.99, 1.14)	0.113	1.01 (0.94, 1.09)	0.911	0.95 (0.90, 1.02)	0.178
Histidine	0.096	*2.03E-05*	0.056	1.16 (1.07, 1.25)*	*2.16E-05*	1.07 (0.99, 1.16)	0.087	0.93 (0.87, 0.99)	0.017
Homoarginine	0.089	*<1.00E-16*	*8.48E-04*	2.09 (1.85, 2.36)*	*<1.00E-16*	1.73 (1.52, 1.98)*	*6.32E-14*	0.83 (0.74, 0.93)	*2.58E-04*
Isoleucine	0.580	*1.95E-04*	0.059	0.94 (0.84, 1.04)	0.316	0.83 (0.74, 0.93)	*3.13E-04*	0.89 (0.81, 0.97)	0.008
Lysine	0.079	*4.85E-04*	0.007	1.11 (1.03, 1.19)	0.003	1.02 (0.94, 1.10)	0.851	0.92 (0.86, 0.98)	0.005
Methionine	0.166	0.011	0.737	1.14 (1.01, 1.28)	0.027	1.02 (0.91, 1.15)	0.892	0.90 (0.81, 1.00)	0.040
Methylarginine	0.005	*6.64E-04*	0.703	0.84 (0.76, 0.94)	*5.27E-04*	0.88 (0.79, 0.98)	0.015	1.04 (0.95, 1.14)	0.544
Ornithine	0.223	*<1.00E-16*	0.065	0.79 (0.71, 0.88)	*2.69E-06*	0.57 (0.51, 0.64)	*3.08E-13*	0.72 (0.66, 0.79)	*1.53E-13*
Phenylalanine	0.035	*8.48E-04*	0.009	1.04 (0.95, 1.14)	0.521	0.92 (0.84, 1.01)	0.090	0.88 (0.82, 0.96)	*6.20E-04*
Proline	0.326	*<1.00E-16*	0.438	0.57 (0.53, 0.61)	*<1.00E-16*	0.54 (0.49, 0.59)	*2.00E-13*	0.95 (0.88, 1.02)	0.167
SDMA	*1.18E-05*	*<1.00E-16*	0.408	0.98 (0.91, 1.05)	0.723	1.37 (1.28, 1.48)*	*2.32E-13*	1.40 (1.32, 1.49)*	*2.48E-13*
Serine	0.028	*1.97E-04*	0.779	0.92 (0.84, 1.02)	0.132	0.84 (0.76, 0.93)	*1.88E-04*	0.91 (0.83, 0.99)	0.022
Taurine	0.040	*2.01E-05*	0.243	1.11 (1.03, 1.20)	0.002	0.99 (0.91, 1.07)	0.924	0.89 (0.83, 0.95)	*7.48E-05*
Threonine	0.003	*8.94E-09*	0.003	1.11 (1.01, 1.21)	0.024	1.28 (1.16, 1.41)*	*1.45E-08*	1.15 (1.07, 1.25)*	*1.03E-04*
Tryptophan	0.027	*6.35E-05*	0.029	1.10 (0.99, 1.23)	0.105	0.92 (0.82, 1.03)	0.186	0.84 (0.76, 0.92)	*4.19E-05*
Tyrosine	0.007	*1.54E-05*	*0.0012*	0.90 (0.84, 0.97)	0.005	0.85 (0.78, 0.92)	*8.42E-06*	0.94 (0.88, 1.01)	0.087
Valine	0.013	*7.88E-13*	*0.0015*	0.93 (0.87, 1.01)	0.076	0.79 (0.73, 0.86)	*2.14E-11*	0.85 (0.80, 0.90)	*1.54E-08*

1 As a Bonferroni correction was used to account for multiple testing, significance was set at *P* < 0.00185 (indicated in *italics*). *Denotes fold changes representing an increase over time. Both ADMA and SDMA were decreased at 34 wk in the +LNS group. Homoarginine, tyrosine, and valine were positively associated with ppBMI. ADMA, asymmetric dimethylarginine; ppBMI, prepregnancy BMI; SDMA, symmetric dimethylarginine.

### Hypertensive disorders of pregnancy

Elevated ADMA concentrations have been previously reported to be associated with pregnancy-related hypertension and preeclampsia. As a result, we investigated incidences of these disorders more broadly in this subcohort and the entire Guatemalan study site. Only 6/134 subjects reported a hypertensive disorder of pregnancy (3 women each in the +LNS and −LNS groups). Statistical analysis was not performed to compare ADMA concentrations in the hypertensive compared with the normotensive subjects due to the small number of subjects involved. The incidence of hypertension in the entire Women First Guatemala study site (*n* = 22) did not vary with supplementation in women who delivered live infants (*n* = 792, data not shown).

### Replication cohort

A total of 179 Pakistani women were included as a replication cohort (Table 1). Similarly to the subject characteristics of the Guatemalan women, subject characteristics of the Pakistani women showed no significant differences between the +LNS and −LNS groups, with the exception of folate supplementation at 34 wk (Table 1). In the Pakistani cohort, unlike the Guatemalan cohort, 95.2% of +LNS subjects received Supplement 2 due to low BMI or insufficient gestational weight gain. There were also significant differences between women from Guatemala and Pakistan regarding ppBMI, maternal height, SES score, mode of delivery, infant sex, and folate supplementation (Table 1). Moreover, these 2 populations have different dietary intake (39).

Despite the differences in subject characteristics and environmental factors between the primary and replication cohorts, 16 of the 23 metabolite concentrations replicated for changes over time (*P* < 0.05), showing similar fold-change patterns over pregnancy. Of these, all but cysteine replicated at *P* < 0.00185 (**Table 3**;**Supplemental Figure 2**). Choline, taurine, and threonine also changed over pregnancy, but with distinct patterns in the 2 populations. Methionine and arginine did not statistically change over pregnancy in either cohort. Only ADMA and glutathione had temporal changes in the Pakistani subjects (*P* < 0.00185) but not in the Guatemalan subjects. The effect of supplementation on ADMA and SDMA concentrations at 34 wk in Guatemala was not replicated in Pakistan. Similar to Guatemala, ppBMI did not significantly influence the relations of time and concentration for any metabolite. The positive association of tyrosine concentration with ppBMI seen in Guatemala was replicated (*P* = 0.0167); isoleucine was positively associated with ppBMI (*P* = 0.0014) in Pakistan but not in Guatemala.

**TABLE 3:** Fold change of log-transformed metabolite concentrations over time with *P* values in the Pakistani replication cohort^1^

				Baseline to 12 wk		Baseline to 34 wk		12 to 34 wk	
Metabolite	Overall supplement *P* value	Overall time *P* value	Overall ppBMI *P* value	Fold change (CI)	*P* value	Fold change (CI)	*P* value	Fold change (CI)	*P* value
ADMA	0.413	<1.00E-16^^^	0.858	0.78 (0.72, 0.84)	5.41E-13	0.83 (0.77, 0.90)	5.22E-08	1.07 (1.00, 1.15)	0.035
Alanine	0.445	*<1.00E-16*	0.048	*0.74 (0.68, 0.80)*	*2.75E-13*	*0.90 (0.83, 0.97)*	*0.005*	1.22 (1.14, 1.31)	4.92E-10
Arginine	0.160	0.002	0.318	1.00 (0.90, 1.11)	1.000	1.13 (1.02, 1.26)	0.017	1.14 (1.03, 1.25)	0.006
Betaine	0.807	*<1.00E-16*	0.495	*0.58 (0.53, 0.64)*	*3.25E-14*	*0.42 (0.38, 0.46)*	*9.48E-13*	*0.72 (0.66, 0.78)*	*7.22E-13*
Choline	0.882	*1.76E-07*	0.218	0.72 (0.61, 0.85)	1.17E-05	0.68 (0.58, 0.81)	6.35E-07	0.95 (0.82, 1.10)	0.677
Citrulline	0.710	*<1.00E-16*	0.681	*0.65 (0.59- 0.71)*	*1.00E-13*	*0.54 (0.49, 0.59)*	*1.21E-12*	*0.83 (0.77, 0.90)*	*4.05E-07*
Creatine	0.800	*<1.00E-16*	0.127	1.05 (0.97, 1.15)	0.317	*1.38 (1.26, 1.50)*	*9.09E-13*	*1.31 (1.21, 1.41)* *	*8.20E-13*
Creatinine	0.631	*<1.00E-16*	0.055	*0.71 (0.66, 0.76)*	*4.01E-13*	*0.79 (0.74, 0.84)*	*9.99E-13*	1.11 (1.04, 1.18)	2.29E-04
Cysteine (total)	0.545	*0.003*	0.400	0.90 (0.82, 0.99)	0.018	*0.88 (0.80, 0.96)*	*0.003*	0.98 (0.90, 1.06)	0.753
Glutamine	0.698	0.736	0.825	*1.02 (0.89, 1.16)*	0.957	0.98 (0.85, 1.12)	0.913	0.96 (0.85, 1.08)	0.723
Glutathione (total)	0.660	1.49E-05 ^^^	0.626	0.88 (0.82, 0.94)	1.63E-05	0.90 (0.84, 0.96)	7.02E-04	1.02 (0.96, 1.09)	0.666
Histidine	0.602	0.234	0.760	1.13 (0.95, 1.35)	0.234	1.11 (0.92, 1.32)	0.385	0.98 (0.83, 1.15)	0.941
Homoarginine	0.163	*1.09E-05*	0.542	*1.30 (1.14, 1.48)* *	*9.45E-06*	*1.25 (1.09, 1.43)* *	*4.16E-04*	0.96 (0.85, 1.08)	0.672
Isoleucine	0.127	*2.55E-08*	0.0014^^^	0.84 (0.76, 0.92)	2.90E-05	*0.79 (0.72, 0.84)*	*3.12E-08*	0.94 (0.87, 1.03)	0.235
Lysine	0.998	0.122	0.256	0.93 (0.86, 1.01)	0.108	0.96 (0.88, 1.04)	0.371	1.02 (0.95, 1.10)	0.746
Methionine	0.360	0.937	0.328	0.99 (0.88, 1.10)	0.960	1.00 (0.89, 1.12)	1.000	1.01 (0.92, 1.12)	0.943
Methylarginine	1.000	*1.61E-07*	0.075	*0.80 (0.71, 0.89)*	*4.04E-06*	0.79 (0.71, 0.88)	1.75E-06	0.99 (0.90, 1.09)	0.980
Ornithine	0.858	*<1.00E-16*	0.359	*0.72 (0.64, 0.81)*	*7.97E-10*	*0.59 (0.52, 0.66)*	*7.30E-13*	*0.81 (0.73, 0.91)*	*2.07E-05*
Phenylalanine	0.784	0.140	0.224	0.94 (0.86, 1.02)	0.168	0.94 (0.87, 1.02)	0.209	1.00 (0.93, 1.08)	0.995
Proline	0.086	*<1.00E-16*	0.431	*0.66 (0.60, 0.72)*	*7.95E-14*	*0.76 (0.69, 0.84)*	*1.88E-10*	1.16 (1.07, 1.26)	1.59E-04
SDMA	0.229	*<1.00E-16*	0.728	0.79 (0.75, 0.85)	2.62E-13	1.05 (0.98, 1.12)	0.184	1.32 (1.25, 1.40)*	*1.04E-12*
Serine	0.832	*1.46E-09*	0.834	0.73 (0.65, 0.82)	7.77E-09	*0.76 (0.67, 0.85)*	*3.35E-07*	1.04 (0.93, 1.16)	0.729
Taurine	0.706	*0.010*	0.823	0.89 (0.80, 1.00)	0.037	0.87 (0.78, 0.98)	0.012	0.98 (0.88, 1.08)	0.872
Threonine	0.434	*3.19E-11*	0.719	0.88 (0.80, 0.98)	0.009	1.16 (1.05, 1.28)*	*1.71E-03*	*1.31 (1.20, 1.44)* *	*2.48E-11*
Tryptophan	0.362	*9.14E-05*	0.466	0.98 (0.90, 1.08)	0.911	0.86 (0.79, 0.95)	7.29E-04	*0.88 (0.81, 0.95)*	*7.99E-04*
Tyrosine	0.525	*2.99E-04*	*0.017*	0.89 (0.81, 0.98)	0.009	*0.86 (0.78, 0.94)*	*2.80E-04*	0.96 (0.89, 1.04)	0.513
Valine	0.809	*1.46E-13*	0.088	0.85 (0.79, 0.91)	7.71E-07	*0.78 (0.72, 0.84)*	*9.76E-13*	*0.92 (0.86, 0.98)*	*0.009*

1 Values in *italics* validate findings seen in the primary Guatemalan cohort, *P* < 0.05. Isoleucine was positively associated with ppBMI, and tyrosine validated findings in Guatemala, *P* < 0.05. *Denotes fold changes representing an increase over time. ^Indicates significant finding in Pakistani, *P* < 0.00185, but not Guatemalan cohort. ADMA, asymmetric dimethylarginine; ppBMI, prepregnancy BMI; SDMA, symmetric dimethylarginine.

## Discussion

This study makes a significant contribution toward our understanding of the effect of a LNS intervention and ppBMI on longitudinal 1C metabolite and AA concentrations from prior to conception through late pregnancy. It is particularly important to explore these effects in populations suffering from growth stunting, obesity, and poor nutrition in order to improve the long-term health of offspring exposed to the triple malnutrition burden in utero. We showed that nearly all of the measured 1C metabolites and AAs significantly changed during pregnancy, with similar patterns between the primary study group and the replication cohort. Neither LNS consumption nor ppBMI significantly modified metabolite concentrations throughout the span of pregnancy.

The temporal concentration changes of many metabolites are similar to those previously reported in healthy pregnant women. For example, we found lower concentrations of betaine (28, 40), alanine (33, 41), citrulline, creatinine, and glutamine (33) at 12 wk compared to before pregnancy. At 34 wk of gestation compared to both preconception and 12 wk, we detected decreases in betaine (40, 42) and valine (27, 31, 43); increases in creatine (44), SDMA (45), and threonine (27, 31, 41); and no statistical changes in the concentrations of arginine (31, 41, 43, 45), glutathione (32), and methionine (31, 46).

Our results contrast with those of previous work for several metabolites, including ADMA (45), choline (28, 40), and isoleucine (31, 41). In other instances, published reports for several metabolites are highly variable, making direct comparisons to our results more complicated. For instance, there is conflicting information regarding the concentration changes of cysteine (27, 31, 32), phenylalanine (27, 41), and taurine (27, 31, 41) toward the end of pregnancy. Mixed results in the literature and between other studies and our findings could reflect differences in subject populations, techniques, or study design, including gestational age at time of blood draw. Other possibilities include fasting at the time of sampling (31, 47, 48) compared with not fasting (33, 34); use of plasma (31, 33, 43), whole blood (34, 47), or DBS cards; age of subjects (43); and/or measurement of metabolites using nuclear magnetic resonance (33) compared with MS (31, 47, 48).

As expected, the replication cohort from Pakistan showed striking differences in subject characteristics with our primary study population in Guatemala including ppBMI. Guatemala is the only Women First Trial site with high rates of maternal obesity (36); therefore, it was not possible to select a replication cohort that matched mean subject ppBMI. Despite these differences, we saw remarkable similarities in 1C metabolite and AA concentration changes from preconception through the third trimester. Sixteen 1C metabolites and AAs with significant changes between time points had the same direction of change in both populations (Table 3; Supplemental Figure 2). The strong commonalities in temporal metabolite patterns suggest a potentially high degree of generalizability among pregnant women, as the patterns were consistent regardless of diet, supplementation, ethnicity, and ppBMI. The biologic mechanisms for and implications of these temporal patterns are not precisely understood. Many of these metabolites are understudied, particularly in the context of pregnancy. Decreased maternal levels may reflect shunting nutrients to the fetus due to higher requirements. For example, betaine is directly involved in homocysteine recycling in the 1C pathway (6, 12, 40) and maternal levels may decrease due to the high rate of DNA methylation in the growing fetus. For other metabolites, fetal need did not appear to be driving concentrations; for example, those for methionine did not change from preconception through gestation.

Three metabolites had distinct patterns in the 2 populations: taurine, an AA not used in protein synthesis but which is important for fetal growth and placental function (49); threonine, an essential AA; and choline, a major component of cell membranes that is critical for fetal brain development (28, 42). Concentrations of all 3 increased in the Guatemala population during the first trimester but decreased in the Pakistan cohort (Supplemental Figure 2). Four other metabolites (glutamine, histidine, lysine, and phenylalanine) had overall time-related patterns that did not replicate. The reasons for these differences could be secondary to variations in underlying dietary intake (39), folate supplementation, or other differences in subject characteristics between the 2 populations, including ethnicity (31, 47). Intakes of choline and betaine were lower in Pakistan, although intakes in both the Pakistani and Guatemalan populations were well below the estimated average requirement (39). Lower protein intake in Pakistan (39) may account for the lower threonine concentration, based on data that threonine decreased in pregnant rats fed a protein-restricted diet (50). Overall, however, the differences were relatively small and may not be clinically relevant.

Contrary to our hypothesis, LNS consumption did not have a measurable impact on most 1C metabolite and AA concentration changes over the course of pregnancy. Only 2 related metabolites, ADMA and SDMA, had lower concentrations at 34 wk in Guatemalan women who received LNS, a finding that was not replicated in the Pakistan cohort. ADMA is a methylated AA that directly inhibits nitric oxide synthase, thereby increasing systemic vascular resistance and blood pressure. SDMA is a stereoisomer of ADMA which does not directly inhibit nitric oxide synthase (51, 52). ADMA, but not SDMA, is elevated during pregnancy in women with preeclampsia, a pattern seen prior to the development of clinical symptoms (30, 51–54). There was no difference in incidence of hypertension during pregnancy in women consuming LNS, making any clinical effect of this reduction unclear. However, the trial was not specifically designed to investigate hypertension or preeclampsia, and clinical data such as blood pressure measurements were not collected, thus limiting interpretation.

Previous reports have demonstrated the influence of diet and supplement intake on some of these metabolites during pregnancy (26–28). One possible reason we did not detect a difference is that that a high portion of the −LNS subjects were consuming additional folate (84.6% in Guatemala). Because homocysteine can be converted back to methionine in a folate-dependent or -independent fashion (12, 20), consumption of folate could be influencing the patterns reported here. However, significantly fewer women in the Pakistani replication cohort consumed additional folate during pregnancy (23.6%), and daily dietary consumption of folate was also lower in Pakistan than in Guatemala (90 compared with 348 μg/d, respectively) (39). The LNS did not contain choline or betaine, which could also account for the absence of statistical differences in these metabolites (28).

The Women First Trial observed increased linear growth in the offspring of women receiving the LNS intervention (36). The mechanisms responsible for increased birth length are not yet known. Only some of the metabolites reported here have been previously studied in relation to fetal growth, primarily birth weight. In pregnancies affected by fetal growth restriction or preeclampsia, maternal ADMA concentrations were inversely correlated with birth weight (55). Maternal betaine concentrations may also be negatively related to birth weight (56), although other investigators have reported no association (57). No relation has been found between birth weight and maternal concentrations of arginine (55), choline (57), creatine (44), or SDMA (55). Because LNS consumption did not have a measurable and consistent impact on metabolite concentrations, it is unlikely that alterations to the 1C pathway are responsible for increased birth length. In subsequent analyses we will investigate whether any metabolite can predict birth weight or length.

Despite covering a wide spectrum of ppBMI (13.8–38.4 kg/m^2^), we did not detect statistically significant differences due to ppBMI in 1C metabolite or AA patterns over the course of pregnancy. Only tyrosine concentrations were positively associated with ppBMI in both cohorts, similar to a previous report (47). Tyrosine is also elevated in obese nonpregnant women (58) and in women with preeclampsia (59). Whether increased tyrosine indicates impaired placental perfusion in obese pregnant women is not currently known. Few studies have investigated the longitudinal influence of ppBMI on metabolite concentrations throughout pregnancy, especially with preconception measurements. Of these, most studies have similarly shown that elevated ppBMI has little effect on 1C metabolites and AAs in pregnant women (44, 47, 48, 60–63). One study that investigated third-trimester AA concentrations showed associations with ppBMI (47); however, this study used self-reported prepregnancy weight data that may have been subject to bias (64).

A limitation of this study is the use of DBS cards instead of serum or plasma samples. The use of DBS cards prevented our ability to reliably measure some critical 1C metabolites known to be influenced by maternal diet, particularly homocysteine, S-adenosyl methionine, S-adenosyl homocysteine, vitamins B6 and B12, and riboflavin (26). DBS cards were used due to their ease of collecting samples in field settings; their cost effectiveness for longitudinal collections; the lack of requirement for trained phlebotomists; and for their ease in shipping. These advantages allowed us to undertake these measurements in 2 understudied populations. Furthermore, DBS cards are widely used for measurement of AAs and other metabolites as part of newborn screening (65, 66) and other studies (67, 68). An additional limitation is that the subjects were not fasting at the time of blood draw. Previous work has shown differences in some metabolites between the fasted and unfasted state (47). However, all women in the study were not fasting, so any effect should be randomly distributed among study groups and should not disproportionately influence our results. Similar studies have likewise used nonfasting samples (32–34).

Despite these limitations, there are many strengths to this study. Most notably, the prospective longitudinal design, including preconception measurements, and targeted quantitative measurements of 1C metabolites and AAs are major strengths. Moreover, evaluation of LNS consumption, the assessment of ppBMI across a wide spectrum, and utilization of a replication cohort diverse from the initial population increase the generalizability of our findings.

In this study, we measured 1C metabolites and AAs longitudinally from preconception through the third trimester and evaluated the effects of LNS and ppBMI in a low- and a middle-income country, where it is increasingly important to address the triple burden of growth stunting, obesity, and micronutrient deficiencies to improve population health. We showed high concordance of most metabolite concentrations over time between the study site and our replication cohort and no significant relations of temporal patterns with LNS consumption or ppBMI. Future work will investigate associations of metabolite concentrations with pregnancy and infant outcomes, including DNA methylation at birth.

## Supplementary Material

nzz132_Supplement_Files

## Notes

Supported by the Bill & Melinda Gates Foundation OPP1055867 (NFK and KMH); the *Eunice Kennedy Shriver* National Institute of Child Health and Human Development and Office of Dietary Supplements U10 HD076474 (KMH and NFK); and the National Institute of Diabetes and Digestive and Kidney Diseases K01 DK109077 (SJB). This publication was also made possible by the University of Florida's Southeast Center for Integrated Metabolomics through grant number U24 DK097209 from the National Institute of Health's Common Fund metabolomics program. The funding bodies had no role in the design of the present study, nor with collection, analysis, and interpretation of data or in writing the manuscript.

Author disclosures: The authors report no conflicts of interest.

Availability of data and material: The metabolomics datasets will be deposited to the University of California San Diego Metabolomics Workbench (www.metabolomicsworkbench.org).

Supplemental Table 1 and Supplemental Figures 1 and 2 are available from the “Supplementary data” link in the online posting of the article and from the same link in the online table of contents at https://academic.oup.com/cdn/.

Abbreviations used: AA, amino acid; ADMA, asymmetric dimethylarginine; DBS, dried blood spot; LME, linear mixed effect; LNS, small-quantity lipid-based nutrition supplement; ppBMI, prepregnancy BMI; SDMA, symmetric dimethylarginine; SES, socioeconomic status; 1C, one carbon.
