# Supplementary material for: Longitudinal Changes of One-Carbon Metabolites and Amino Acid Concentrations during Pregnancy in the Women First Maternal Nutrition Trial
Source: Curr Dev Nutr. 2019 Nov 18;4(1):nzz132. doi: 10.1093/cdn/nzz132 (PMC7064164; doi:10.1093/cdn/nzz132)
Supplement: nzz132_Supplement_Files [file nzz132_supplement_files.zip › Supplementary Figures.pptx]

## Slide 1
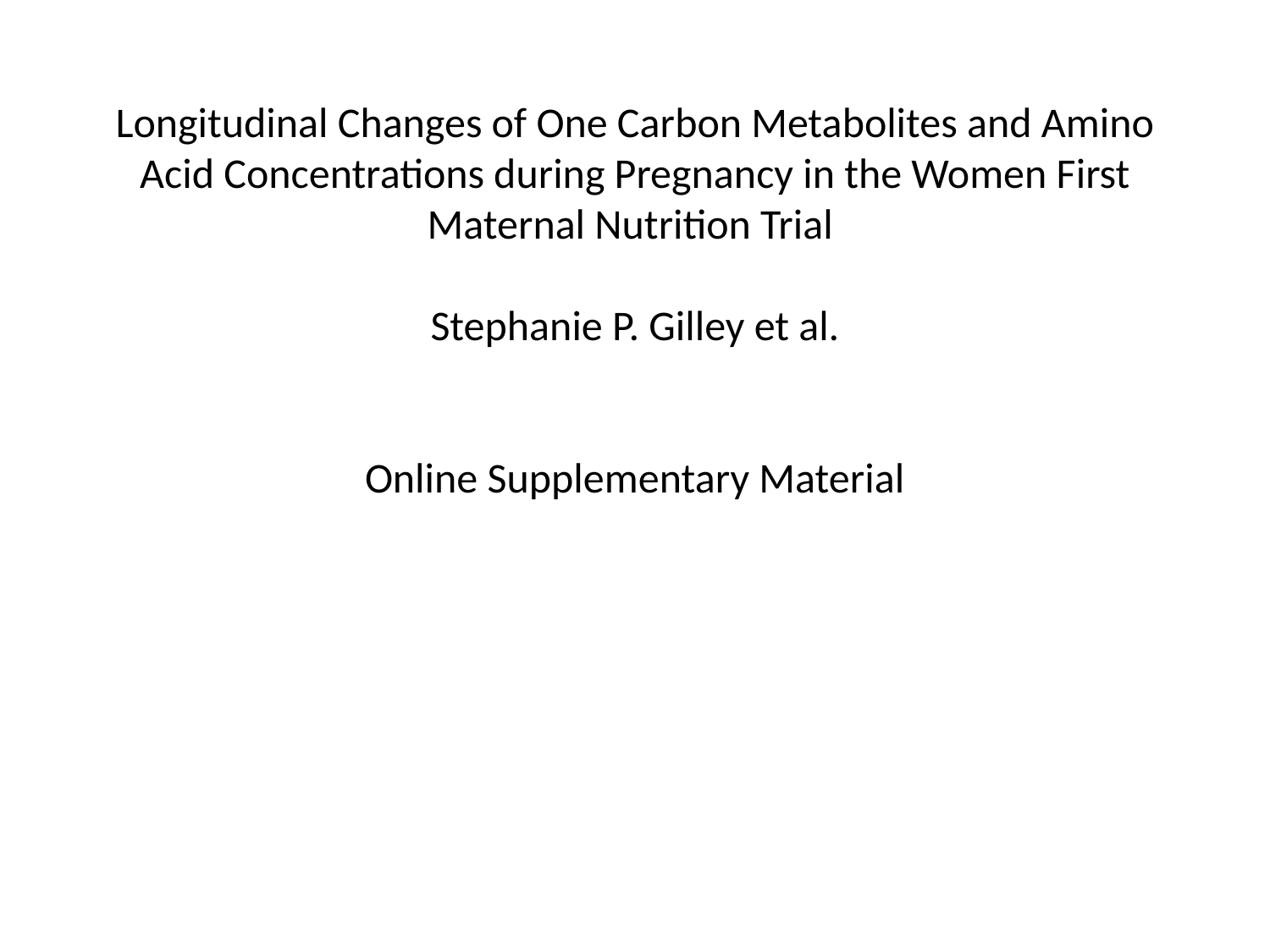

# Longitudinal Changes of One Carbon Metabolites and Amino Acid Concentrations during Pregnancy in the Women First Maternal Nutrition Trial Stephanie P. Gilley et al. Online Supplementary Material

## Slide 2
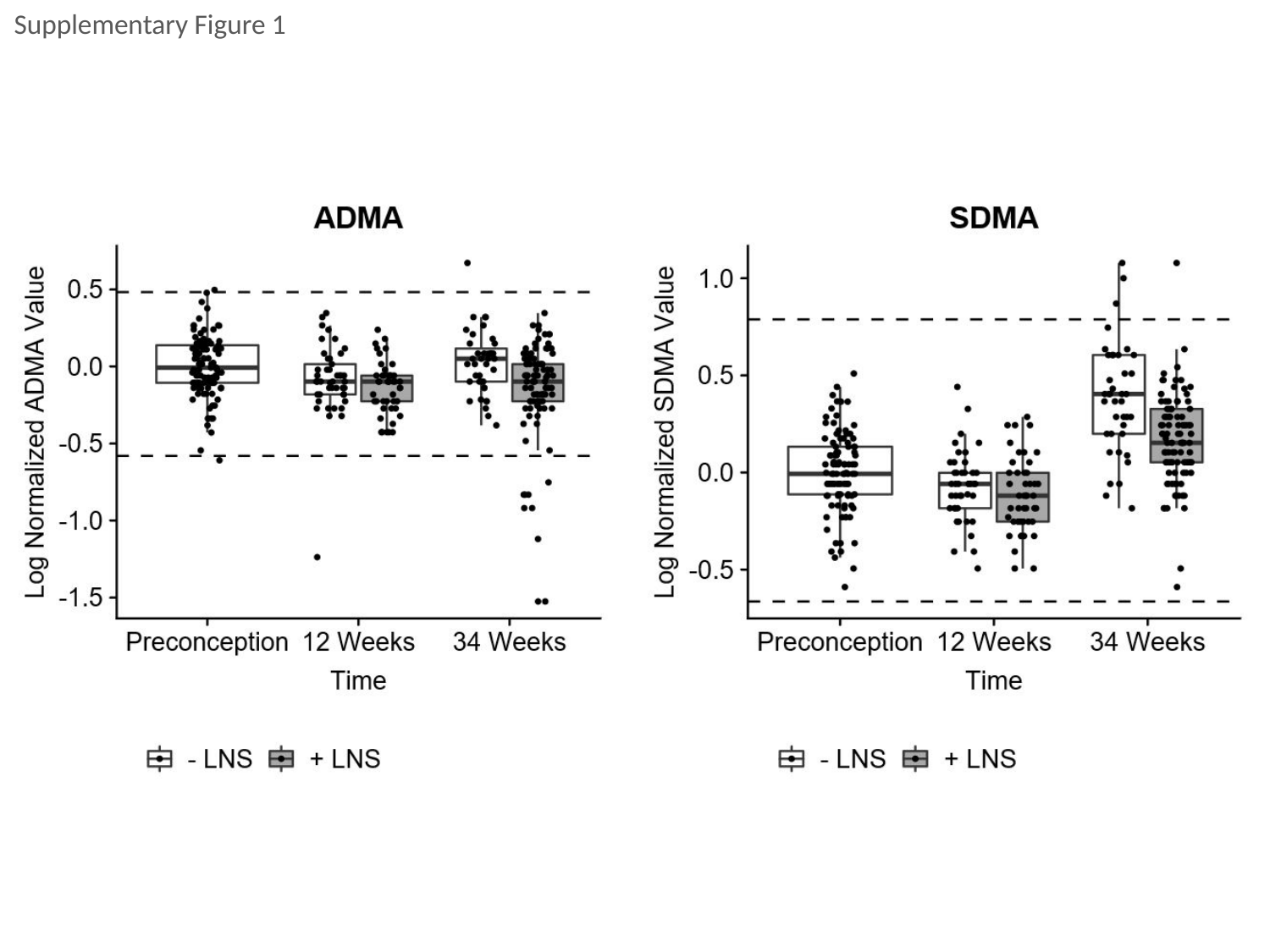

Supplementary Figure 1

## Slide 3
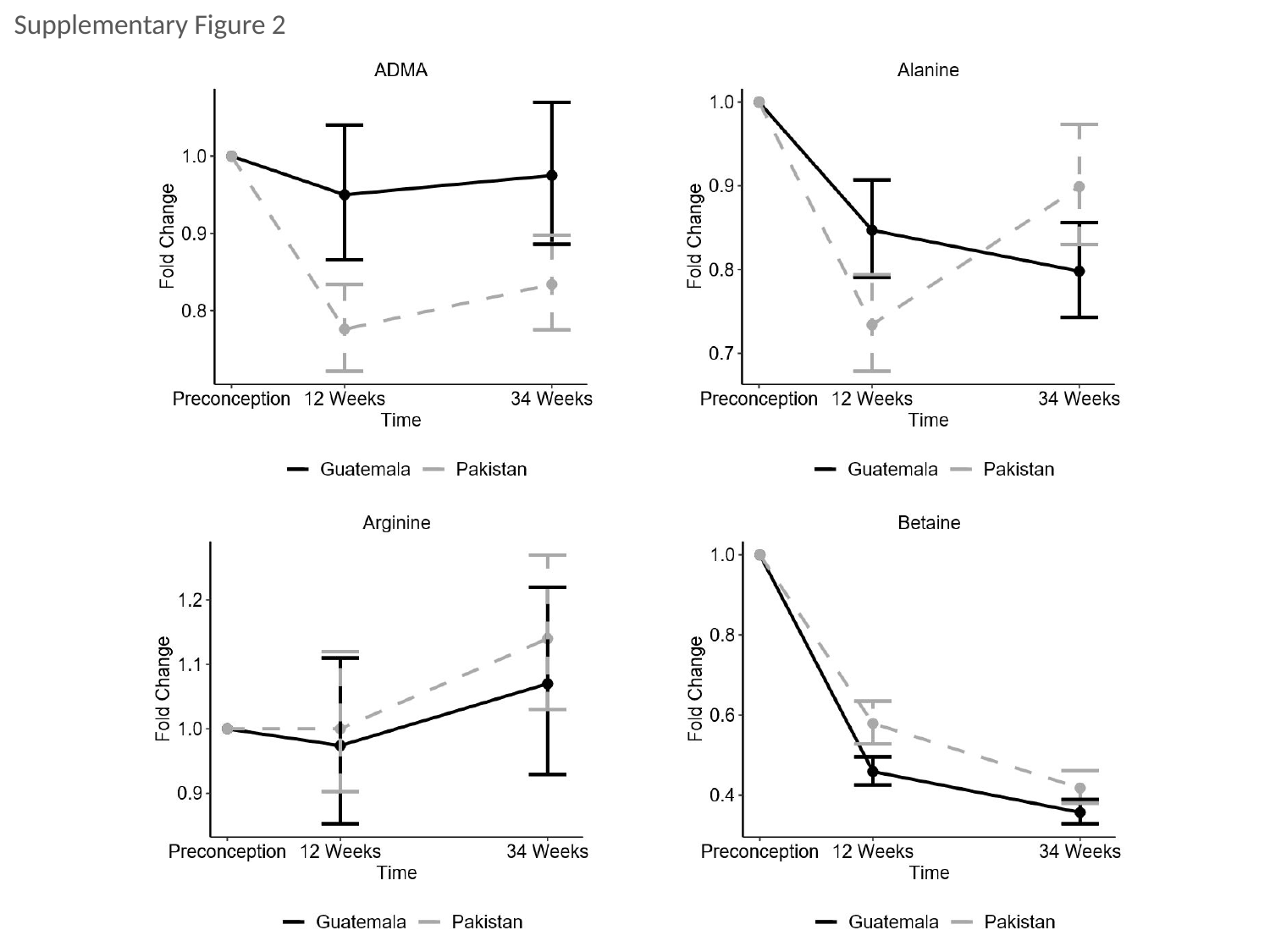

Supplementary Figure 2

## Slide 4
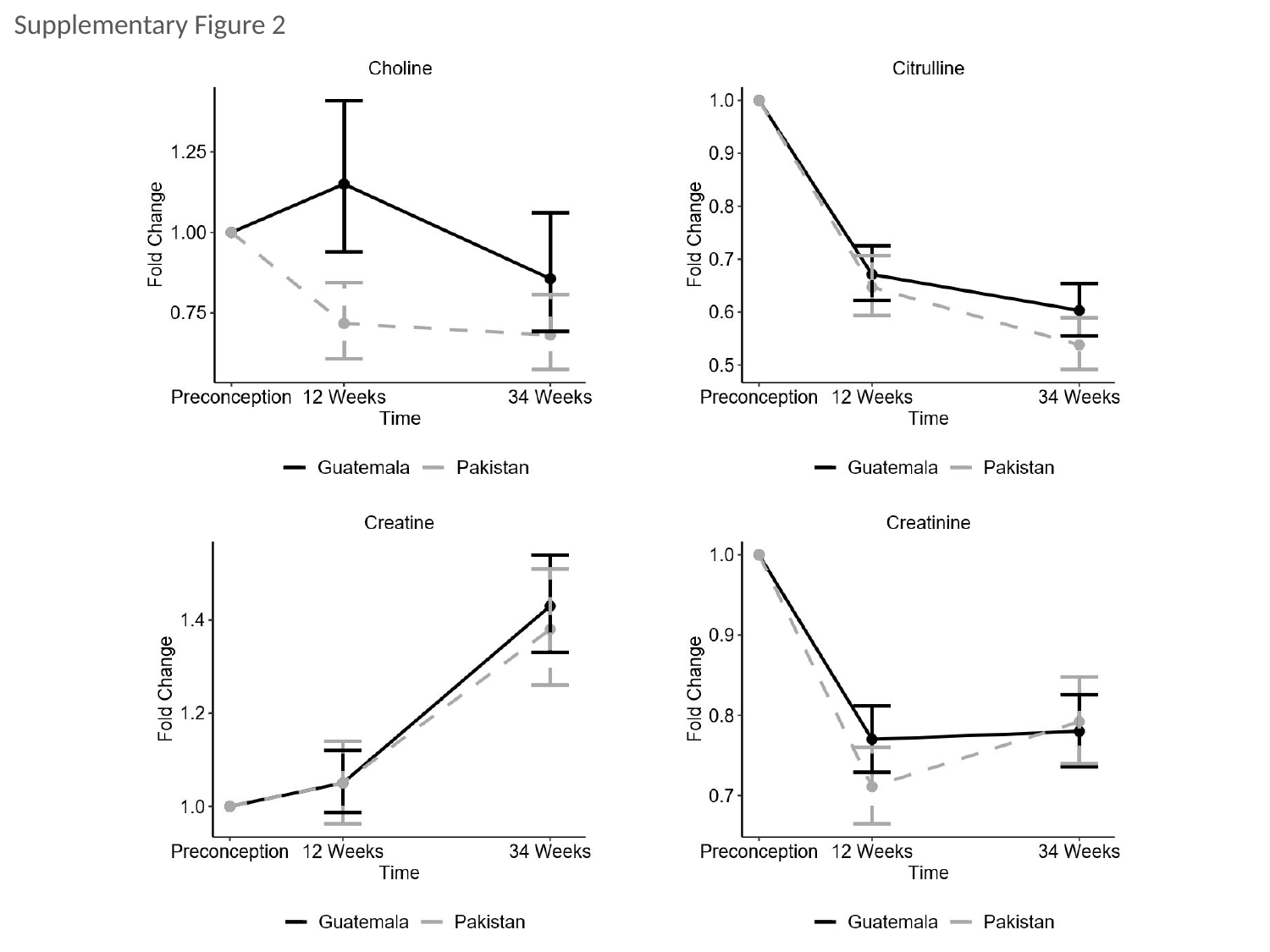

Supplementary Figure 2

## Slide 5
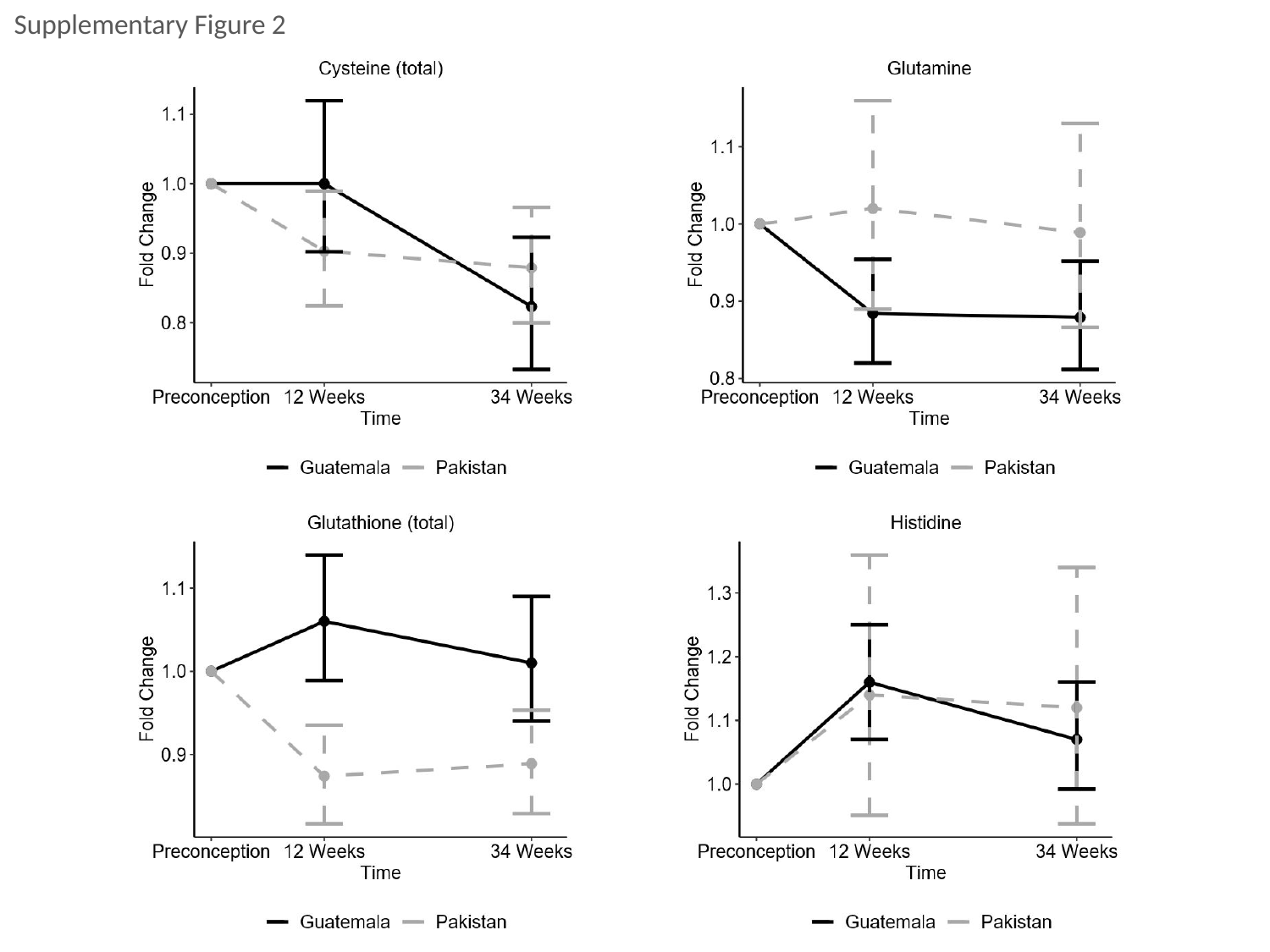

Supplementary Figure 2

## Slide 6
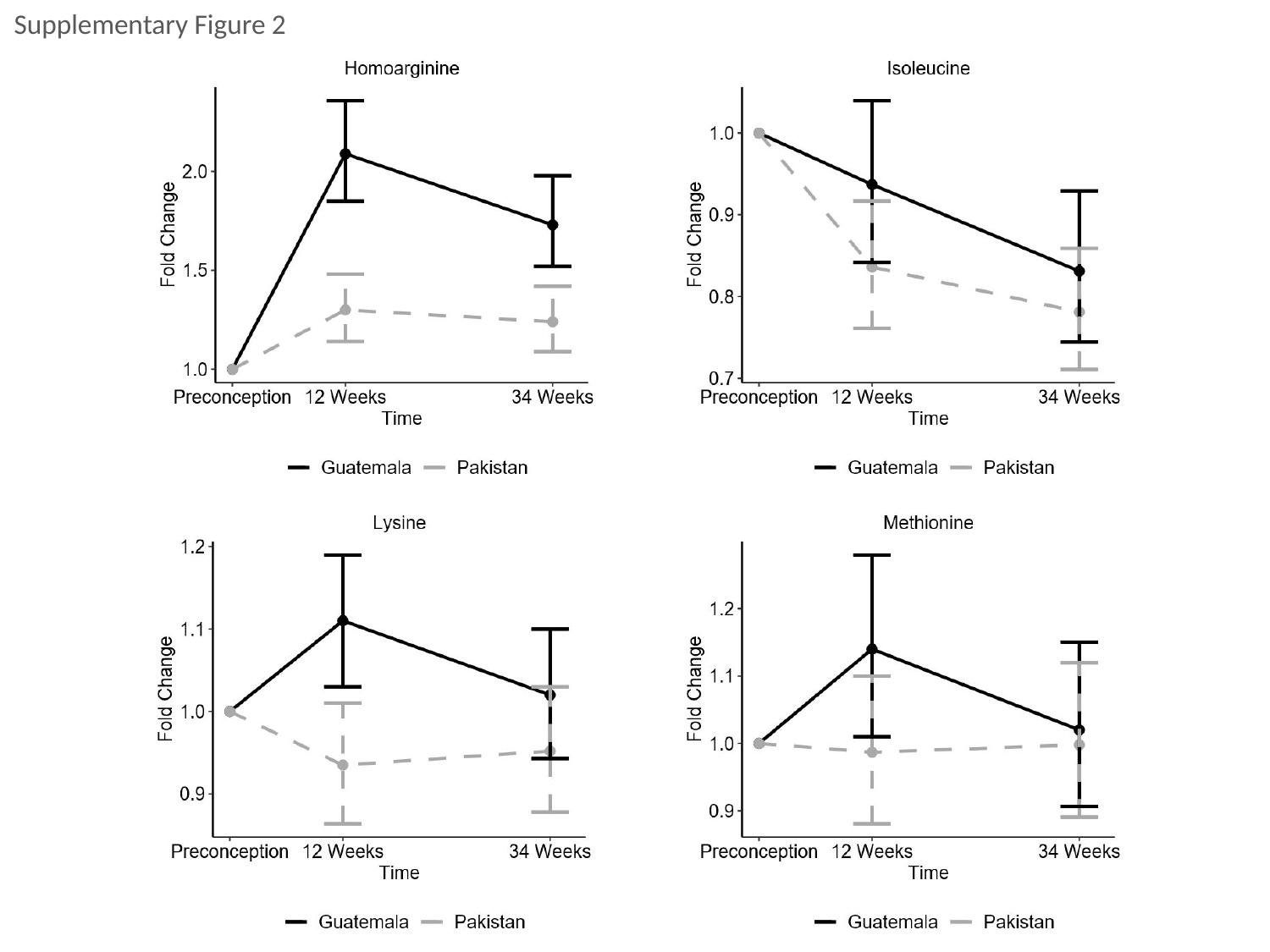

Supplementary Figure 2

## Slide 7
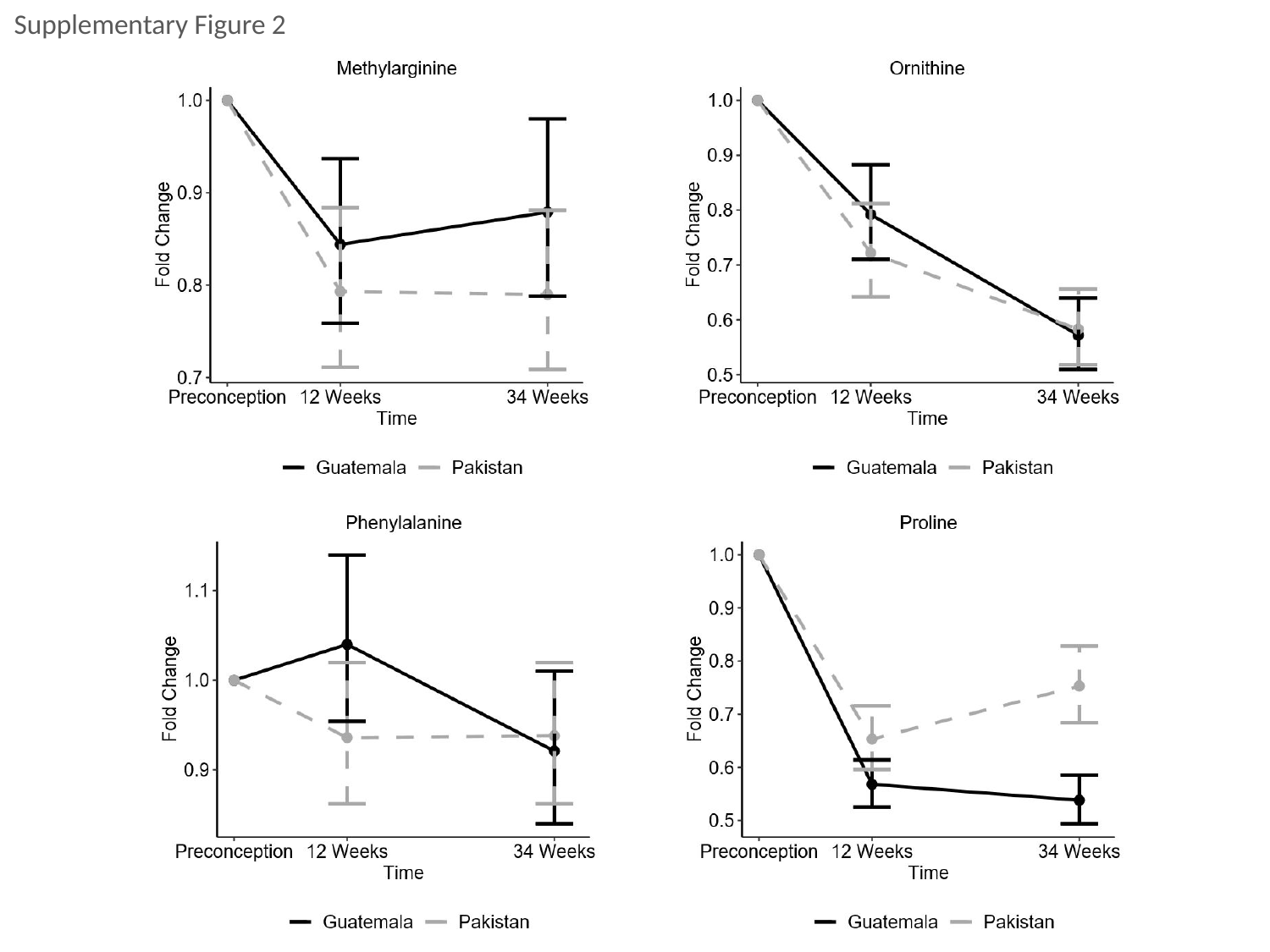

Supplementary Figure 2

## Slide 8
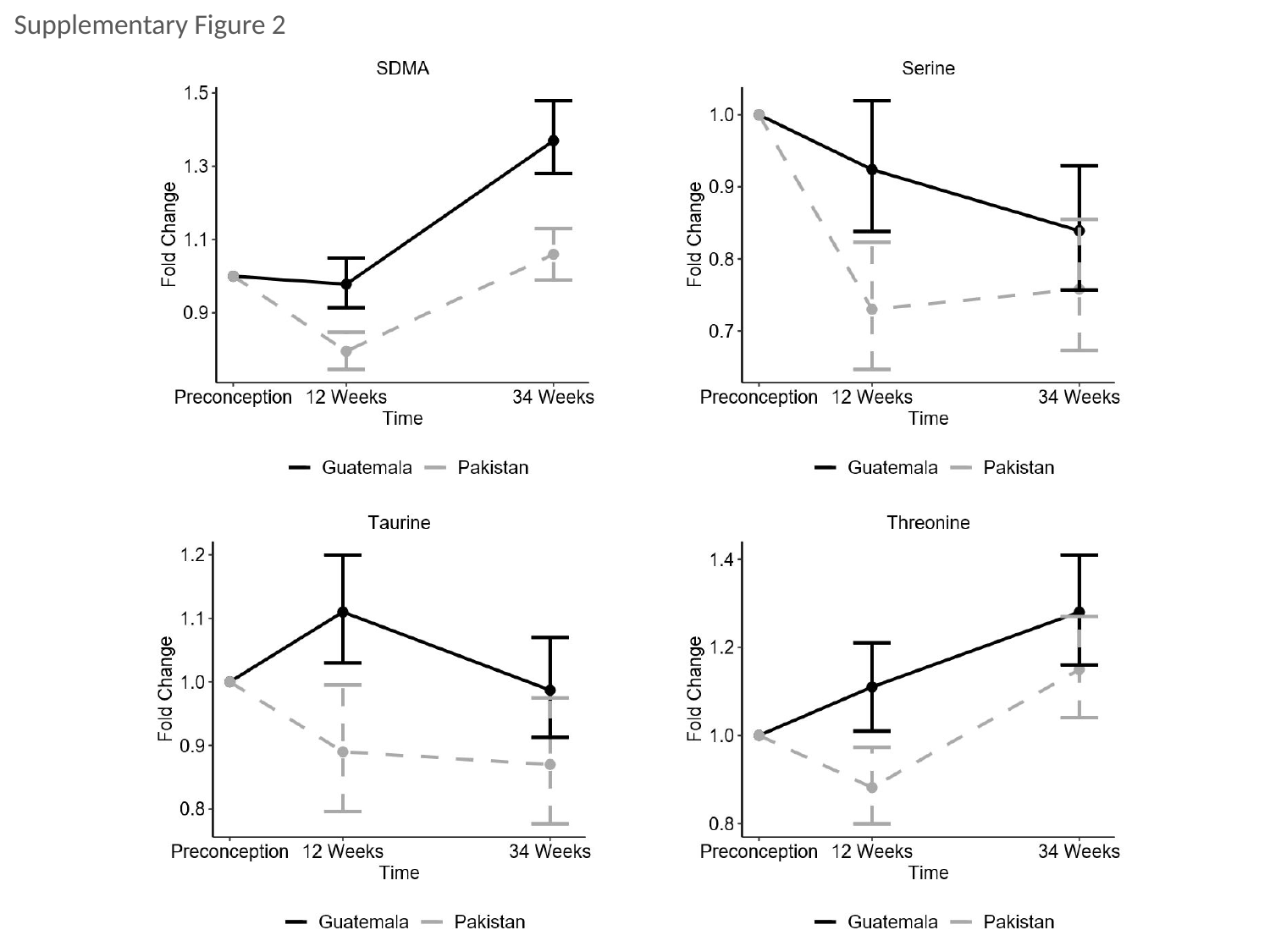

Supplementary Figure 2

## Slide 9
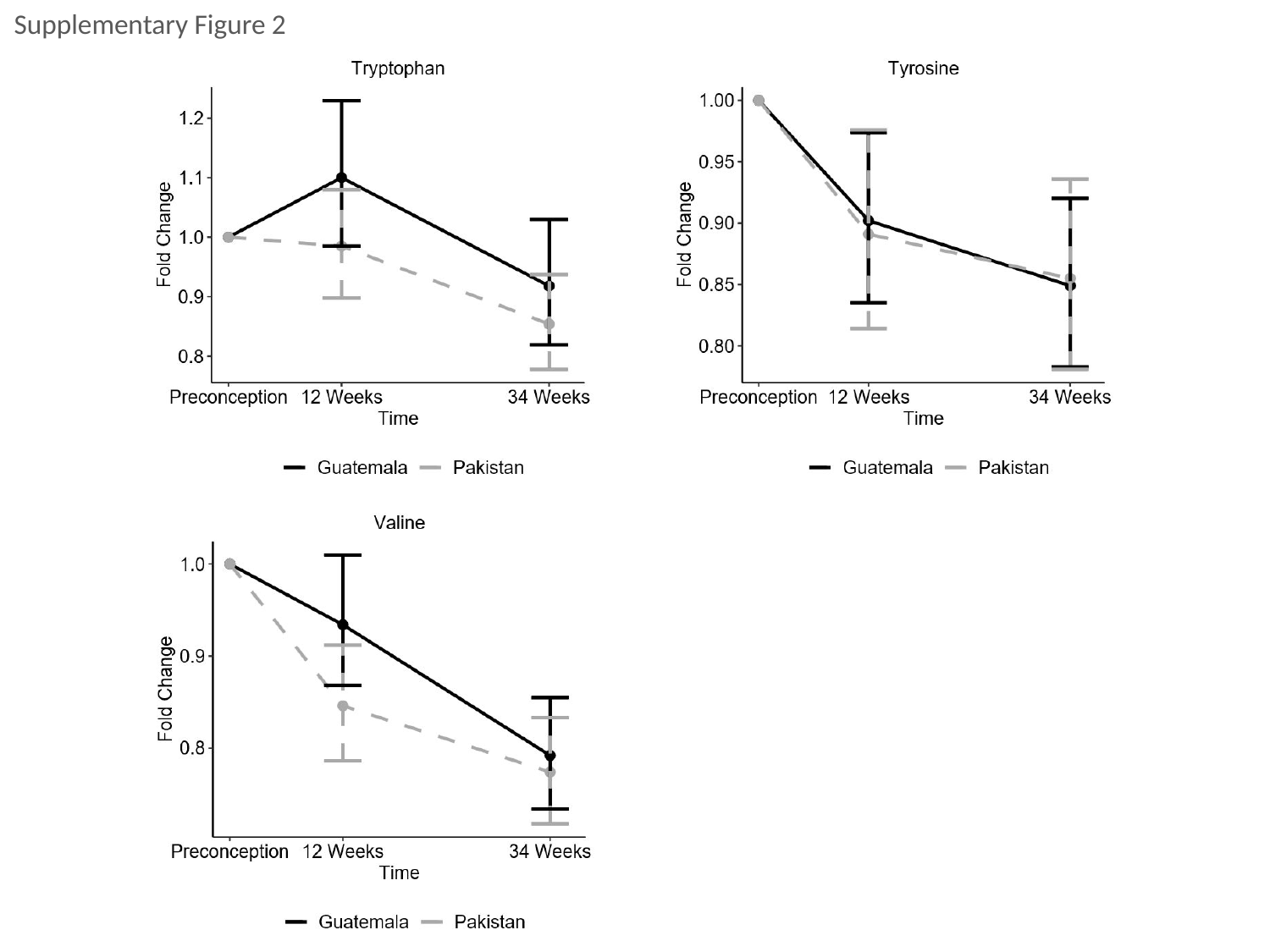

Supplementary Figure 2
