# Supplementary material for: Longitudinal Changes of One-Carbon Metabolites and Amino Acid Concentrations during Pregnancy in the Women First Maternal Nutrition Trial
Source: Curr Dev Nutr. 2019 Nov 18;4(1):nzz132. doi: 10.1093/cdn/nzz132 (PMC7064164; doi:10.1093/cdn/nzz132)
Supplement: nzz132_Supplement_Files [file nzz132_supplement_files.zip › Supplementary tables.docx]

**Supplementary Material**

| **Nutrient** | **Amount** | **Nutrient** | **Amount** | **Nutrient** | **Amount** |
| --- | --- | --- | --- | --- | --- |
| Energy | 118 kcal | Iron | 20 mg | Thiamine (B1) | 2.8 mg |
| Protein | 2.6 g | Magnesium | 65 mg | Vitamin A | 800 μg |
| Fat | 10 g | Manganese | 2.6 mg | Vitamin B12 | 5.2 μg |
| Linoleic Acid | 4.59 g | Niacin | 36 mg | Vitamin B6 | 3.8 mg |
| α-Linolenic Acid | 0.59 g | Pantothenic acid (B5) | 7 mg | Vitamin C | 100 mg |
| Calcium | 280 mg | Phosphorous | 190 mg | Vitamin D2 | 1000 IU |
| Copper | 4 mg | Potassium | 200 mg | Vitamin E | 20 mg |
| Folate | 400 μg | Riboflavin (B2) | 2.8 mg | Vitamin K | 45 μg |
| Iodine, *μg* | 250 μg | Selenium | 130 μg | Zinc | 15 mg |

**Supplementary Table 1: Composition of the small-quantity lipid-based nutrition supplement (LNS).** The supplement is a multi-micronutrient fortified lipid-based supplement from Nutriset which was modified for use in pregnant and lactating women. It includes soybean and peanut extract, dried skimmed milk, sugar, maltodextrin stabilizers, and emulsifiers in addition to the micronutrients and polyunsaturated lipids. For more details, see Reference (35).

**Supplementary Figure 1:** Log-normalized asymmetric dimethylarginine (ADMA) and symmetric dimethylarginine (SDMA) concentrations over time by LNS status in the primary Guatemalan cohort. White boxes show blood draws from unsupplemented time points (-LNS) while shaded boxes are blood draws taken while the mother had been consuming LNS for greater than 12 weeks. Boxes shows 25^th^-75^th^ percentiles with the line showing mean concentrations (50^th^ percentile). Whiskers represent 5^th^ and 95^th^ percentiles. Dashed lines represent 1.5 times the interquartile range which were used to define outliers. Exclusion of outliers did not alter the findings, and they were therefore included in the final analysis.

**Supplementary Figure 2:** Fold changes of log-transformed metabolite concentrations over the course of pregnancy for the Guatemala (black solid line) and Pakistan (gray dashed line) cohorts. +LNS and -LNS groups were merged for these figures as there was not a consistent effect of the supplement for any metabolite in both population cohorts. A fold change of 1.0 represents no change from preconception. Error bars show 95% confidence intervals.
